# Artificial intelligence diagnostics for bladder tumor identification and grade prediction depend on narrow band imaging cystoscopy

**DOI:** 10.1016/j.isci.2025.114309

**Published:** 2025-12-03

**Authors:** Yinchao Wang, Hao Liang, Yaozhong Zhang, Wenqiang Qi, Guangping Wu, Xiaoyi Zhang, Chuanpeng Li, Shouzhen Chen, Jun Chen, Benkang Shi

**Affiliations:** 1 Department of Urology, Qilu Hospital of Shandong University, Jinan 250012, Shandong, China; 2 Department of Urology, Qilu Hospital of Shandong University (Qingdao), Qingdao, China; 3 Department of Urology, Rizhao Central Hospital, Rizhao, China; 4 Albert Einstein College of Medicine - Jacobi Medical Center, New York, NY, USA; 5 MsunHealth Technology Group Co., Ltd., No. 1237 Yingxiu Road, Jinan, Shandong 250101, China; 6 Department of Urology, Qilu Hospital of Shandong University, Jinan 250012, Shandong, China

**Keywords:** Oncology, medical imaging, artificial intelligence applications

## Abstract

The effective treatment of bladder cancer depends on early evaluation through cystoscopy. Given the clinical importance of distinguishing the tumor grade, we report the application of the AI-assisted NBI Cystoscopy Diagnostic System (AINCDS). The AINCDS consists of (1) dual-channel feature extraction module, (2) lesion segmentation module based on feature pyramids, and (3) a multi-task classification module. AINCDS achieved an accuracy for identifying bladder cancer of 0.919 (95% CI = 0.896 to 0.938). For the prediction of tumor grade, the accuracy was 0.764 (95% CI = 0.714 to 0.810). The AINCDS demonstrates similar ability comparable to urologists with over 10 years’ experience. With the assistance of AINCDS, the tumor grade prediction accuracy of urologists with 1–3 years’ experience improved from 0.667 to 0.793. AINCDS can assist in the diagnosis of bladder cancer and prediction of tumor grade, offering the potential to improve the accuracy of lesion assessment and reduce the workload of urologists.

## Highlights

• AINCDS identifies bladder cancer and predicts tumor grade with NBI
• AINCDS proposes a dual-channel feature extraction strategy
• AINCDS incorporates a feature-sharing strategy to achieve computational efficiency

## Graphical abstract

**Figure:**
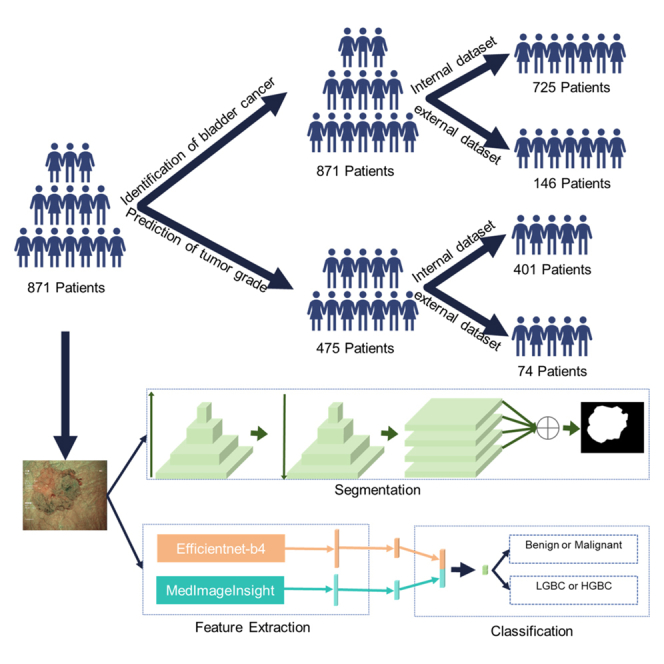

## Introduction

Bladder cancer is the most common malignant tumor in the urinary system and is also one of the most prevalent cancers worldwide. According to the Global Cancer Statistics 2022, bladder cancer ranks ninth in new cases, accounting for 3.1% of all new cancer diagnoses,^1^ showing an increasing trend compared to 3% in 2020.^2^ Cystoscopy is a critical diagnostic and therapeutic tool for bladder cancer, widely used in early diagnosis, preoperative assessment, biopsy, electroresection, and postoperative follow-up.^3^ To improve the accuracy of evaluations, various image enhancement technologies have been applied in recent years.^4^^,^^5^^,^^6^^,^^7^^,^^8^ Narrow band imaging (NBI) is an optical image enhancement technology that filters light into two frequencies, 415 nm and 540 nm. The 415 nm light is blue, and the 540 nm light is green. Both are easily absorbed by hemoglobin, making blood vessels more visible.^9^^,^^10^ The enhanced vascular imaging in NBI images aids urologists in lesion evaluation during cystoscopy. Existing studies have shown that NBI has significant advantages over white light imaging (WLI) in the diagnosis and treatment of bladder cancer.^11^^,^^12^^,^^13^^,^^14^^,^^15^^,^^16^ However, the diagnosis of tumors under NBI currently relies solely on the subjective judgment of the physician. Our previous research described the possible morphological features of tumors under NBI,^17^ but it was abstract, making it difficult for less experienced doctors.

Cystoscopy can only provide a rough judgment of the benign or malignant nature of the tumor. Even experienced urologists often find it challenging to determine the tumor grade solely through cystoscopy. We know that high-grade bladder cancer has a higher risk of muscle invasion. Therefore, during surgery, it is necessary to resect a deeper and broader area, and in some cases, multiple biopsies are required.^18^ Thus, a thorough preoperative assessment of tumor grading is significant for formulating a precise surgical plan.

In recent years, the rise of artificial intelligence (AI) in the endoscopic field has made AI-assisted diagnostic systems a trend. In 2020, Shaoxu Wu and his colleagues developed the Cystoscopy Artificial Intelligence Diagnostic System (CAIDS) using neural networks, achieving over 95% accuracy in identifying bladder cancer under WLI.^19^ In 2022, J. W. Yoo et al., through deep learning and using the RGB method, also achieved 94.1% accuracy in identifying bladder cancer under WLI.^20^ A. T. Lenis and M. S. Litwin emphasized that in clinical practice, the prediction of tumor grade and muscle invasion is more meaningful.^21^ J. W. Yoo et al. also achieved accuracy of 65.1% and 56.2% under WLI and NBI in predicting tumor grade. However, the images used for training might be insufficient for deep learning, with only 1,800 NBI images and 3,768 WLI images used for tumor color information collection, and the images were highly selected images with good tumor delineation identified by both clinicians and AI.^20^

In our study, we used a new framework for feature extraction, lesion segmentation, and multi-task classification to build the AINCDS. On one hand, we trained AINCDS to delineate lesions. When the AINCDS achieved a dice score coefficient (DSC) of over 0.800 in comparison with urologist, we considered it capable of effectively recognizing lesions. On the other hand, we used NBI images delineated by urologists as training dataset for AINCDS to identify bladder cancer and predict tumor grade. Ultimately, through internal and external validation, we confirmed AINCDS’ capability in lesion recognition, malignancy assessment, and tumor grade classification.

## Results

**Figure 1:**
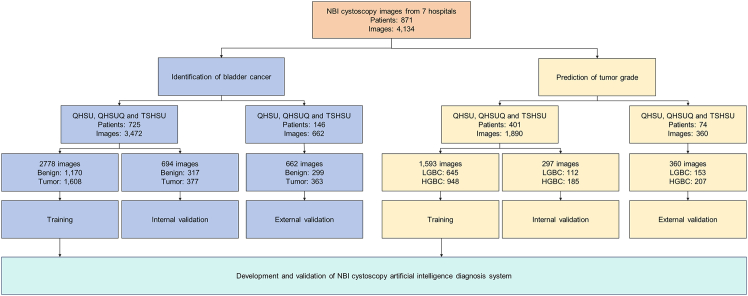
Development and validation of a cystoscopy artificial intelligence diagnosis system QHSU, Qilu Hospital of Shandong University; QHSUQ, Qilu Hospital of Shandong University (Qingdao); TSHSU, The Second Hospital of Shandong University; TFAHSFMU, The First Affiliated Hospital of Shandong First Medical University; TCCH, Taian City Central Hospital; SPH, Shandong Provincial Hospital; YYH, Yantai Yuhuangding Hospital.

In total, we collected 4,114 images from 871 patients across 7 centers. Among these, 362 cases of benign lesions were represented by 1,786 images, and 509 cases of malignant lesions by 2,348 images. There were 183 cases of low-grade tumors with 889 images, and 292 cases of high-grade tumors with 1,361 images. The detailed data composition for each center is shown in Tables 1 and 2. For internal validation, we used data from Qilu Hospital of Shandong University (QHSU), Qilu Hospital of Shandong University (Qingdao) (QHSUQ), and The Second Hospital of Shandong University (TSHSU). For external validation, we used data from The First Affiliated Hospital of Shandong First Medical University (TFAHSFMU), Taian City Central Hospital (TCCH), Shandong Provincial Hospital (SPH), and Yantai Yuhuangding Hospital (YYH). Due to the small sample sizes from SPH and YYH, we did not separately discuss their results in the external validation section. Details are shown in Figure 1.

**Table 1:** Characteristics of enrolled patients

Characteristic	QHSU (*n* = 611)		QHSUQ (*n* = 69)		TSHSU (*n* = 45)		TFAHSFMU (*n* = 71)		TCCH (*n* = 60)		SPH (*n* = 8)		YYH (*n* = 7)	
	Benign	Tumor	Benign	Tumor	Benign	Tumor	Benign	Tumor	Benign	Tumor	Benign	Tumor	Benign	Tumor
Patients No.	251	360	26	43	14	31	34	37	35	25	0	8	2	5
Age, Median (IQR), years	61.4 (56.0–70.0)	65.6 (56.0–70.0)	59 (50.3–67)	64.3 (59.5–70.5)	60.8 (50.5–69.5)	70.1 (65.5–74.5)	57.3 (50.3–66.5)	65.7 (61.0–72.0)	63 (58.0–72.5)	70.5 (63.0–77.0)	NA	66.6 (58.5–73.5)	61.0 (NA)	62.8 (54.0–71.0)
Sex, No.														
Male	178	309	23	38	11	24	23	28	27	23	0	8	2	3
Female	73	51	3	5	3	7	11	9	8	2	0	0	0	2
Grade No.														
Low grade	–	127	–	9	–	14	–	18	–	8	–	5	–	2
High grade	–	208	–	26	–	17	–	19	–	17	–	2	–	3
Ungraded	–	25	–	8	–	0	–	0	–	0	–	1	–	0

Characteristics of enrolled patients.

IQR, interquartile range; QHSU, Qilu Hospital of Shandong University; QHSUQ, Qilu Hospital of Shandong University (Qingdao); TSHSU, The Second Hospital of Shandong University; TFAHSFMU, The First Affiliated Hospital of Shandong First Medical University; TCCH, Taian City Central Hospital; SPH, Shandong Provincial Hospital; YYH, Yantai Yuhuangding Hospital.

**Table 2:** Characteristics of enrolled images

Characteristic	QHSU (*n* = 3,094)		QHSUQ (*n* = 250)		TSHSU (*n* = 128)		TFAHSFMU (*n* = 352)		TCCH (*n* = 256)		SPH (*n* = 22)		YYH (*n* = 32)	
	Benign	Tumor	Benign	Tumor	Benign	Tumor	Benign	Tumor	Benign	Tumor	Benign	Tumor	Benign	Tumor
Images No.	1,364	1,730	92	158	31	97	152	200	141	115	0	22	6	26
Grade No.														
Low grade	–	656	–	31	–	49	–	84	–	37	–	15	–	17
High grade	–	1,007	–	99	–	48	–	116	–	78	–	4	–	9
Ungraded	–	67	–	28	–	0	–	0	–	0	–	3	–	0

Characteristics of enrolled images.

IQR, interquartile range; QHSU, Qilu Hospital of Shandong University; QHSUQ, Qilu Hospital of Shandong University (Qingdao); TSHSU, The Second Hospital of Shandong University; TFAHSFMU, The First Affiliated Hospital of Shandong First Medical University; TCCH, Taian City Central Hospital; SPH, Shandong Provincial Hospital; YYH, Yantai Yuhuangding Hospital.

### Artificial intelligence-assisted narrow band imaging cystoscopy diagnostic system recognizes lesions effectively

**Figure 2:**
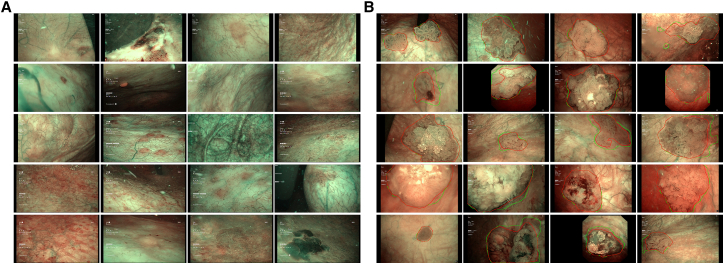
Examples of cases used in our study (A) Examples of benign. (B) Examples of malignancy. The red lines were delineated by urologists, and the green lines were predicted by the AINCDS.

Through the dual-channel feature extraction module and lesion segmentation module, the AINCDS-segmented areas showed good overlap with the areas segmented by urologists. In the internal validation set, the DSC was 0.883, and the intersection over union (IOU) was 0.819. In the external validation set, the DSC was 0.834, and the IOU was 0.740 (Table 3). Some segment examples are shown in Figure 2. Both internal and external validations proved that the system could recognize lesions effectively.

**Table 3:** AINCDS performance in images segmentation

Dataset	DSC	IOU	Recall	Precision
Internal validation set	0.883	0.819	0.899	0.904
exterbal validation set	0.834	0.74	0.902	0.822

AINCDS performance in images segmentation.

DSC, dice score coefficient; IOU, intersection over union.

### Artificial intelligence-assisted narrow band imaging cystoscopy diagnostic system identifies bladder cancer accurately

The AINCDS demonstrated good performance in identifying bladder cancer. In the internal validation set, the accuracy was 0.919 (95% CI = 0.896 to 0.938). The sensitivity was 0.912 (95% CI = 0.879 to 0.939), specificity was 0.927 (95% CI = 0.893 to 0.953), positive predictive value (PPV) was 0.937 (95% CI = 0.907 to 0.960), negative predictive value (NPV) was 0.899 (95% CI = 0.861 to 0.930), and the F1 score was 0.925 (95% CI = 0.901 to 0.942). The external validation results were similar to the internal validation, with all metrics exceeding 0.900 (Table 4).

**Table 4:** AINCDS performance in the identification of bladder cancer

Datasets	Accuracy (95%CI)	Sensitivity (95%CI)	Specificity (95%CI)	PPV (95%CI)	NPV (95%CI)	F1 Score (95%CI)
internal validation set	0.919(0.896–0.938)	0.912(0.879–0.939)	0.927(0.893–0.953)	0.937(0.907–0.960)	0.899(0.861–0.930)	0.925(0.901–0.942)
external validation set	0.931(0.908–0.949)	0.937(0.906–0.959)	0.923(0.887–0.951)	0.937(0.906–0.959)	0.923(0.887–0.951)	0.937(0.915–0.954)
TFAHSFMU	0.918(0.884–0.944)	0.920(0.873–0.954)	0.915(0.859–0.954)	0.934(0.890–0.964)	0.897(0.839–0.940)	0.927(0.894–0.951)
TCCH	0.941(0.905–0.967)	0.939(0.879–0.975)	0.943(0.891–0.975)	0.931(0.869–0.970)	0.950(0.900–0.980)	0.935(0.896–0.961)
SPH	1.000(0.852–1.000)	1.000(0.846–1.000)	1.000(0.025–1.000)	1.000(0.846–1.000)	1.000(0.025–1.000)	1.000(0.852–1.000)
YYH	0.939(0.798–0.993)	1.000(0.868–1.000)	0.714(0.290–0.963)	0.929(0.765–0.991)	1.000(0.478–1.000)	0.963(0.798–0.993)

AINCDS performance in the identification of bladder cancer.

PPV, positive predictive value; NPV, negative predictive value; CI, confidence intervals; TFAHSFMU, The First Affiliated Hospital of Shandong First Medical University; TCCH, Taian City Central Hospital; SPH, Shandong Provincial Hospital; YYH, Yantai Yuhuangding Hospital.

**Figure 3:**
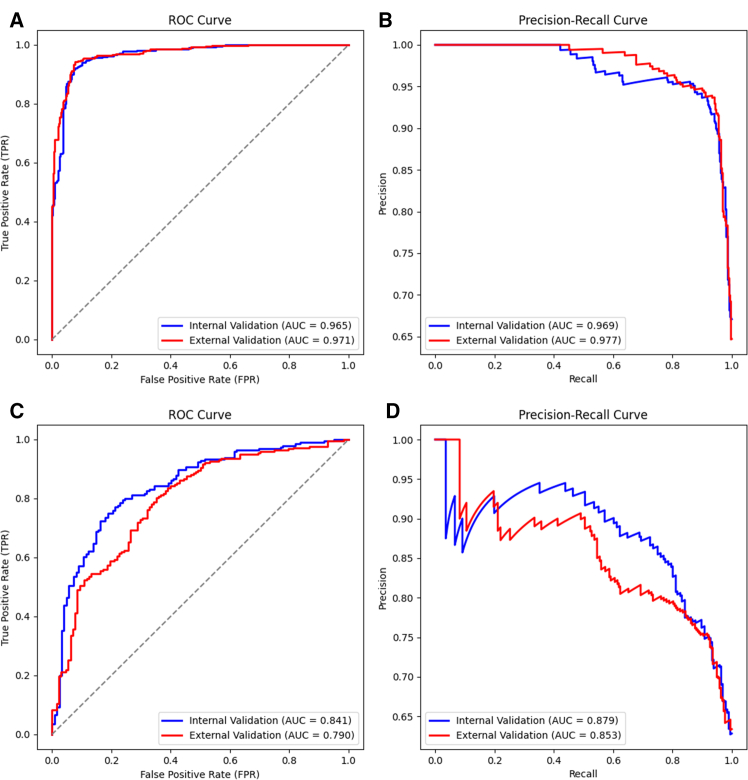
Performance of AINCDS in identifying bladder cancer and predicting tumor grade The ROC curves were generated by plotting sensitivity against specificity. The PR curves were generated by plotting recall (also known as sensitivity) against precision (also known as the positive predictive rate). (A) ROC curves in identifying bladder cancer. (B) PR curves in identifying bladder cancer. (C) PR curves in predicting tumor grade. (D) PR curves in predicting tumor grade. ROC, receiver operating characteristic; PR, precision-recall; AUC, areas under the curves.

To provide a more intuitive display of its performance, we plotted the receiver operating characteristic (ROC) curve and precision-recall (PR) curve. Both curves showed satisfactory results, with the areas under the curves (AUCs) for internal validation being 0.965 and 0.969. For the external validation, the AUC for all curves also exceeded 0.950 (Figures 3A and 3B).

### Artificial intelligence-assisted narrow band imaging cystoscopy diagnostic system predicts tumor grade effectively

In predicting tumor grade, the AINCDS achieved an accuracy of 0.764 (95% CI = 0.714 to 0.810) in the internal validation set. The sensitivity was 0.832 (95% CI = 0.772 to 0.881), which was higher than the specificity of 0.656 (95% CI = 0.564 to 0.739). The relatively high sensitivity helps reduce the underestimation of lesions. The PPV and NPV were similar, at 0.795 (95% CI = 0.733 to 0.848) and 0.708 (95% CI = 0.615 to 0.790). The F1 score was 0.813 (95% CI = 0.764 to 0.853). The external validation results showed no significant difference from the internal validation (Table 5).

**Table 5:** AINCDS performance in the prediction of tumor grade

Datasets	Accuracy (95%CI)	Sensitivity (95%CI)	Specificity (95%CI)	PPV (95%CI)	NPV (95%CI)	F1 Score (95%CI)
internal validation set	0.764 (0.714–0.810)	0.832 (0.772–0.881)	0.656 (0.564–0.739)	0.795 (0.733–0.848)	0.708 (0.615–0.790)	0.813 (0.764–0.853)
external validation set	0.749 (0.699–0.793)	0.894 (0.846–0.932)	0.500 (0.410–0.590)	0.753 (0.696–0.804)	0.736 (0.630–0.824)	0.818 (0.770–0.855)
TFAHSFMU	0.802 (0.737–0.857)	0.967 (0.918–0.991)	0.467 (0.337–0.600)	0.787 (0.712–0.849)	0.875 (0.710–0.965)	0.868 (0.804–0.909)
TCCH	0.689 (0.598–0.771)	0.805 (0.703–0.884)	0.432 (0.271–0.605)	0.759 (0.655–0.844)	0.500 (0.319–0.681)	0.781 (0.687–0.845)
SPH	0.550 (0.315–0.769)	1.000 (0.398–1)	0.438 (0.198–0.701)	0.308 (0.091–0.614)	1.000 (0.590–1)	0.471 (0.231–0.685)
YYH	0.714 (0.513–0.868)	0.700 (0.348–0.933)	0.722 (0.465–0.903)	0.583 (0.277–0.848)	0.812 (0.544–0.960)	0.636 (0.406–0.785)

AINCDS performance in the prediction of tumor grade.

PPV, positive predictive value; NPV, negative predictive value; CI, confidence intervals; TFAHSFMU, The First Affiliated Hospital of Shandong First Medical University; TCCH, Taian City Central Hospital; SPH, Shandong Provincial Hospital; YYH, Yantai Yuhuangding Hospital.

In internal validation, the AUC for the ROC and PR curves was 0.841 and 0.879, both greater than 0.800. The external validation also produced satisfactory results, with the AUC for the ROC curve being 0.790 and for the PR curve being 0.853 (Figures 3C and 3D.).

### Artificial intelligence-assisted narrow band imaging cystoscopy diagnostic system exhibited good performance, assisting the urologist

**Figure 4:**
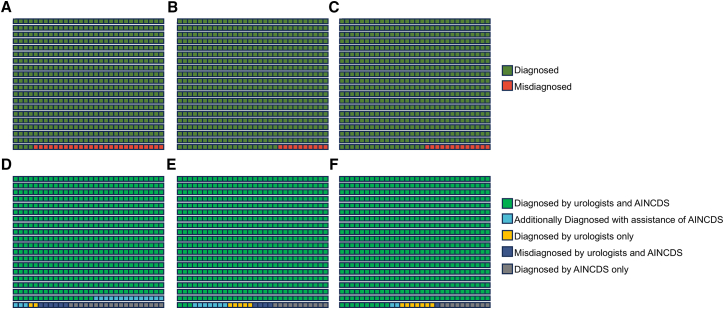
AINCDS assisting urologists in identifying bladder cancer (A, B, and C) represent the identification results made by urologists with 1–3 years, 4–10 years, and over 10 years of experience, respectively, without the assistance of AINCDS. (D, E, and F) represent the identification results made by urologists with 1–3 years, 4–10 years, and over ten years of experience, respectively, with the assistance of AINCDS.

**Figure 5:**
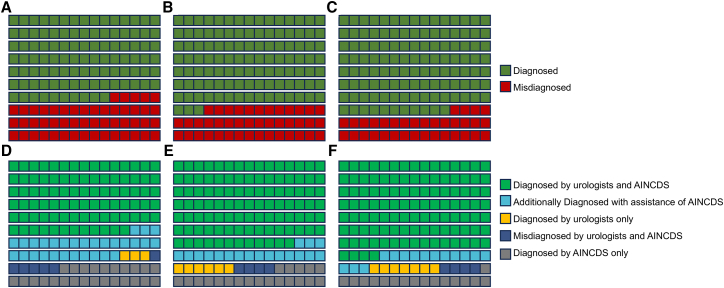
AINCDS Assisting Urologists in predicting tumor grade (A, B, and C) represent the prediction results made by urologists with 1–3 years, 4–10 years, and over 10 years of experience, respectively, without the assistance of AINCDS. (D, E, and F) represent the prediction results made by urologists with 1–3 years, 4–10 years, and over ten years of experience, respectively, with the assistance of AINCDS.

In identifying bladder cancer, urologists demonstrated a high accuracy rate, regardless of the presence of AINCDS assistance (Figure 4). AINCDS exhibited good performance in assisting in tumor grade prediction. The accuracy for urologists with 1–3 years of experience increased from 0.667 to 0.79. For urologists with 4–10 years of experience, the accuracy rate rose from 0.720 to 0.84. Urologists with over ten years of experience saw an increase in accuracy from 0.773 to 0.87 (Figure 5).

## Discussion

**Figure 6:**
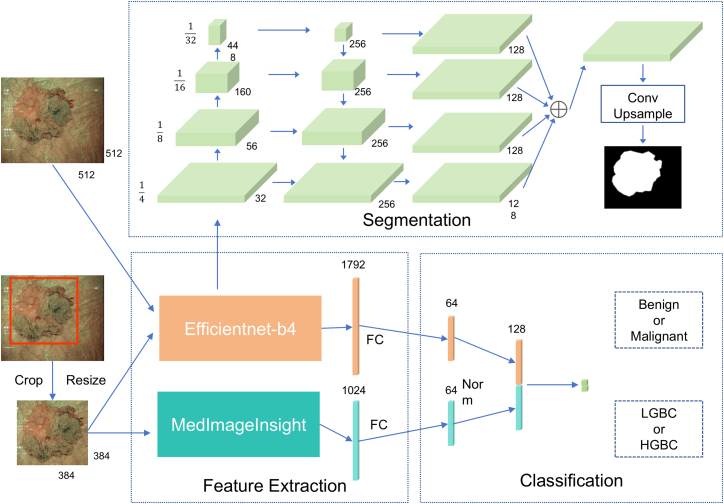
The framework of the AINCDS (1) Dual-channel feature extraction module: integrates general visual features with medically specific features. (2) Lesion segmentation module: A lesion segmentation module based on feature pyramids. (3) Multi-task classification module: Performs L2 normalization and concatenation of dual-channel features, followed by multi-task classification based on the fused features.

We developed AINCDS, which consists of (1) a dual-channel feature extraction module, (2) a lesion segmentation module, and (3) a multi-task classification module (Figure 6). In the dual-channel feature extraction module, EfficientNet-B4^22^ and MedImageInsight^23^ are responsible for feature extraction, integrating both general visual features and medical-specific features. The lesion segmentation module uses EfficientNet-B4 generates a binary segmentation mask with a size of 512 × 512 pixels. The multi-task module locates the lesion using the binary segmentation mask and then extracts features of the lesion from the feature maps obtained by the dual-channel feature extraction module. For feature fusion, the features from both channels are L2-normalized and concatenated into a 128-dimensional joint feature vector. Finally, depending on the task, the fused features are used for the classification of benign/malignant lesion or high/low-grade tumors (Figure 6).

AINCDS has three innovative aspects within its algorithmic framework. First, we propose a dual-channel feature extraction strategy to enhance the representation capability for complex lesions. This strategy not only leverages the strengths of large-scale pre-trained models on generic images but also optimizes for the characteristics of medical imaging, effectively improving diagnostic accuracy and enhancing the model’s generalization ability. In comparison to the baseline models UNet24 and DeepLabV325, the dual-feature extraction network framework facilitates the AINCDS in more effectively diagnosing bladder cancer (Table S1). Ablation experiments reveal that the dual-feature extraction network offers considerable advantages in both the diagnosis of bladder cancer and the prediction of tumor grade (Tables S2 and S3). Additionally, AINCDS employs a feature pyramid segmentation network based on the U-Net architecture, which merges multi-scale features layer by layer to augment the model’s capability in recognizing lesion areas. This method improves adaptability to lesions of varying sizes, ensuring more precise segmentation results and providing reliable information about lesion regions for subsequent classification tasks. Lastly, AINCDS incorporates a feature-sharing strategy that enables the segmentation and classification modules to share EfficientNet-B4 as the feature extraction network, achieving computational efficiency through synergistic collaboration. The segmentation module utilizes EfficientNet-B4 to extract multi-scale features for lesion localization, while the classification module reuses the trained encoder component to extract features from the lesion area for cancer grading. This shared mechanism not only reduces redundant computations and enhances inference efficiency but also maintains feature consistency between the segmentation and classification tasks, thereby improving overall diagnostic performance.

Cystoscopy directly influences subsequent treatment and prognosis. However, lesion evaluation heavily depends on the experience of urologists. The sensitivity and specificity of WLI cystoscopy in identifying bladder cancer are only 0.848 (95% CI = 0.803 to 0.885) and 0.870 (95% CI = 0.831 to 0.90), and rely on the urologist’s experience.^15^ In recent years, NBI in cystoscopy has shown clear advantages over WLI cystoscopy. Currently, experienced urologists using NBI cystoscopy to identify bladder cancer have a specificity of 0.74 (95% CI: 0.63–0.82), which is similar to WLI cystoscopy (0.74, 95% CI: 0.64–0.83). However, NBI cystoscopy has a higher sensitivity of 0.96 (95% CI: 0.92–0.98), which is significantly better than WLI cystoscopy’s 0.75 (95% CI: 0.70–0.79).^16^ Urologists can easily find and assess lesions with NBI, but as a new technology, urologists need time to accumulate experience. Our research has developed an AI-assisted NBI cystoscopy diagnostic system. This innovative system aims to advance the dissemination and standardization of NBI technology, offering a solution to numerous clinical challenges associated with inaccurate tumor assessments, particularly by less experienced clinicians.

A. T. Lenis and M. S. Litwin have suggested that the prediction of tumor grade is more clinically meaningful.^21^ Currently, NBI cystoscopy shows a clear advantage over WLI cystoscopy in diagnosing non-muscle-invasive bladder cancer, with odds ratios (ORs) for Ta stage, T1 stage, and carcinoma *in situ* (CIS) being OR = 4.18 (95% CI = 1.80 to 9.71), OR = 3.39 (95% CI = 1.33 to 8.66), and OR = 5.38 (95% CI = 3.25 to 8.90). However, it does not show an advantage in diagnosing muscle-invasive bladder cancer (OR = 3.19, 95% CI = 0.12 to 84.43).^16^ Our preliminary research revealed that tumor grading can be effectively differentiated based on the imaging characteristics observed in NBI cystoscopy. Building on these findings, we have further developed an AI-assisted NBI diagnostic system to enhance the accuracy of tumor grading.

We believe AINCDS has great clinical potential. First, it can accurately locate lesions, with an internal validation DSC of 0.883, helping urologists easily find lesions during cystoscopy. Second, it shows good performance in identifying bladder cancer and predicting tumor grade, with internal validation accuracies of 0.919 (95% CI = 0.896 to 0.938) and 0.764 (95% CI = 0.714 to 0.810). In the internal validation, the AUC of ROC and PR curves for identifying bladder cancer was greater than 0.95, and for predicting tumor grade, was greater than 0.8, indicating good performance in both accuracy and detection effectiveness, which confirms AINCDS could provide valuable reference for urologists during cystoscopy. Lastly, it is trained to depend on NBI images, and with the increasing adoption of NBI, its application is anticipated to expand further.

With the growing application of AI in the medical field, AI-assisted cystoscopy diagnosis continues to evolve. In 2020, Shaoxu Wu et al. developed CAIDS, which achieved an accuracy of over 95% in identifying bladder cancer based on WLI images.^19^ Shaoxu Wu et al.’s CAIDS has already performed well in identifying bladder cancer. However, there is still limited research on the AI-assisted prediction of tumor grade. A. T. Lenis and M. S. Litwin emphasized that the identification of tumor grade holds greater clinical significance.^21^ In 2022, J. W. Yoo et al. analyzed WLI and NBI cystoscopy images using RGB methods. Due to the wider spectrum of WLI, WLI images contained more RGB information, resulting in higher accuracy. The accuracy of identifying bladder cancer under WLI and NBI was 99.2% and 84.9%, respectively, while the accuracy of predicting tumor grade was 65.1% for WLI and 56.2% for NBI.^20^ The grade prediction accuracy is lower than AINCDS.

In 2022, J. W. Yoo’s AI lesion delineation achieved a DSC of 0.747. They selected only those samples exhibiting high consistency in tumor recognition between the urologists and AI for color feature acquisition and training/validation, ultimately training the AI on only 1,281 NBI images, which may have been too few for the method they used.^20^ We used a more efficient framework and used more data for training. Ultimately, AINCDS achieved a higher DSC of 0.883. We trained and validated it with unfiltered images, and the internal validation accuracy for predicting tumor grade reached 0.764.

In our investigation of the auxiliary role of AINCDS, both AINCDS and urologists exhibited strong performance in diagnosing bladder cancer (Figure 4). Given that all urologists had a high baseline accuracy in bladder cancer diagnosis, AINCDS did not demonstrate a significant auxiliary effect. However, regarding the prediction of tumor grade, we found that AINCDS had a similar ability compared to urologists with over 10 years of experience (0.764 vs. 0.773). Notably, AINCDS provided greater assistance to less experienced urologists; the accuracy of urologists with 1–3 years of experience improved from 0.667 to 0.793. For those with 4–10 years of experience, accuracy increased from 0.720 to 0.840. Urologists with over 10 years of experience also benefited from the assistance of AINCDS, achieving an accuracy of 0.867 (Figure 5).

We have developed an AI-assisted diagnostic system based on NBI imaging, called AINCDS. This system completes lesion segmentation through a U-shaped encoder-decoder architecture combined with a feature pyramid network. Furthermore, it incorporates a dual-channel feature fusion network that integrates the general visual model EfficientNet-B4 with the medical-specific embedding network MedImageInsight, effectively enhancing the representation capability for complex lesions. AINCDS not only accurately diagnoses bladder cancer but also assists in predicting the tumor grade. Looking ahead, this system shows promising application prospects in cystoscopy diagnostic systems and transurethral resection surgical systems.

### Limitations of the study

AINCDS achieved good accuracy in predicting tumor grade, but we acknowledge some shortcomings. First, while we trained AINCDS using images from 871 patients, images from the same patient may have similar characteristics. Although we used image enhancement techniques, we hope to include more data for training and validation in the future to obtain better and more convincing results. Second, in identifying bladder cancer, our diagnostic accuracy is slightly lower than that of Shaoxu Wu and J. W. Yoo’s studies. The RGB method used by J. W. Yoo highly depends on the accuracy of AI-calibrated lesion delineation, and since we have a superior DSC, we are considering using RGB methods to analyze WLI images to identify bladder cancer in the future, then using NBI images to predict tumor grade, enabling more precise lesion assessment. Thirdly, in our preliminary study, the performance of diagnosis of bladder cancer under WLI and NBI was similar; however, NBI demonstrated a significantly superior performance in predicting tumor grade compared to WLI. Consequently, we have only reported the results of NBI. It is important to note that in clinical practice, urologists usually utilize a combination of WLI and NBI to comprehensively assess the tumor. Therefore, in future applications, we will integrate WLI and NBI as complementary diagnostic tools to better align with clinical realities.

## Resource availability

### Lead contact

Further information and requests for resources should be directed to and will be fulfilled by the lead contact, Benkang Shi (bkang68@sdu.edu.cn.).

### Materials availability

This study did not generate new unique reagents.

### Data and code availability

• The image data reported in this study cannot be deposited in a public repository because of the hospital regulation restrictions and patient privacy concerns. The data reported can be shared by the lead contact upon reasonable request. Clinical and demographic details of all participants are shown in Data S1.
• All related codes are showed in Zip S1 and Zip S2 (https://github.com/YinchaoWang/AINCDS-final)
• Any additional information required to reanalyze the data reported in this work article is available from the lead contact upon request.

## Acknowledgments

This work was supported by the Qingdao Science and Technology Demonstration and Guidance Special Fund for the Benefit of the People under Grant [No. 21-1-4-rkjk-7-nsh] and the Olympus Corporation. Independently Cultivate Innovative Teams of Jinan, Shandong Province (No.202228081). The funder played no role in study design, data collection, analysis and interpretation of data, or the writing of this article.

## Author contributions

B.S., J.C., and S.C. are responsible for conceptualization, data curation, funding acquisition, project administration, supervision, validation, and writing-review and editing. Y.W., H.L., Y.Z., W.Q., and X.Z. are responsible for formal analysis, investigation, methodology, software, resources, visualization, and writing-original draft. G.W. and C.L. are responsible for methodology and software. All authors read and approved the final article.

## Declaration of interests

The authors declare no competing interests.

## STAR★Methods

### Key resources table

**Table:** 

REAGENT or RESOURCE	SOURCE	IDENTIFIER
**Software and algorithms**		

Torch	2.0.0+cu117	https://pytorch.org/
Torchvision	0.15.1+cu117	https://github.com/pytorch/vision
Timm	1.0.12	https://github.com/rwightman/pytorch-image-models
Segmentation_models.pytorch	N/A	https://github.com/qubvel-org/segmentation_models.pytorch
Python	3.8.17	https://www.python.org/downloads/release/python-3817/

### Experimental model and subject details

#### Ethics approval and consent to participate

This study was approved by the institutional ethics committee (Medical Ethics Committee of Qilu Hospital of Shandong University (Qingdao), approval number: KYLL-2021010). Informed consent form is available at https://github.com/YinchaoWang/AINCDS-final.

#### Images collection and annotated dataset

We collected NBI cystoscopy data from QHSU between December 2017 and December 2023, as well as from QHSUQ, TSHSU, TFAHSFMU, TCCH, SPH, and YYH from January 2022 to June 2024. Images with blurriness, insufficient brightness, metastatic tumors, no lesions, or those without pathological confirmation were excluded. Ultimately, a total of 4,114 NBI cystoscopy images from 871 patients were included. Of these, 816 patients had images collected during the cystoscopy procedure, while 55 patients had images extracted from cystoscopy videos. Among the included patients, 509 were diagnosed with bladder cancer, accounting for 2,348 images, while 362 were diagnosed with benign lesions, comprising 1,786 images. The group with low-grade tumor consisted of 183 patients and 889 images, and the high-grade tumor included 292 patients and 1,361 images. All diagnoses were confirmed through cystoscopy sample biopsy or surgical electrocautery sample biopsy. The grade of bladder cancer was determined based on pathology with standard of 2004 WHO Classification of Classification of Tumor of Urinary System and Male Genital Organs. In cases where there was a discrepancy between cystoscopy sample biopsy and surgical electrocautery biopsy results, the latter was used as the definitive diagnosis. The images included were annotated by a professional urologist using Labelme to delineate the lesion boundaries, and these annotations were verified by another experienced urologist.

We initially confirmed the pathology using the patients’ formal clinical pathology reports. These reports are generated by certified hospital pathologists and subsequently reviewed by senior pathologists. The pathology of the cases included in our study was re-evaluated by a professional pathologist, and any cases with uncertainties were excluded from the study.

### Method details

#### AI algorithm

The automatic diagnostic system developed in this study consists of three core modules, as illustrated in Figure 6: (1) Dual-channel feature extraction module, integrating both general visual features and medical-specific features; (2) Lesion segmentation module based on a feature pyramid; and (3) Multi-task classification module. The system takes NBI cystoscopy images as input and sequentially performs lesion segmentation and localization, cancer screening, and malignant grading diagnosis tasks.

#### Dual-channel feature extraction module

The feature extraction module adopts a parallel dual-network architecture, integrating both general visual features and medical-specific features. The EfficientNet-B4 backbone network is initialized with ImageNet pre-trained weights and employs a compound scaling strategy to balance network depth, width, and resolution. This network accepts 512 × 512 input images and outputs hierarchical representations containing multi-scale features. The medical-specific network, MedImageInsight, is a lightweight, general-purpose medical image embedding model designed to support cross-task and cross-modal medical image analysis and generation tasks. The model’s image encoder is based on the DaViT (Dual Attention Vision Transformer) architecture, which can extract high-dimensional image features from a multi-modal shared embedding space. Both feature extraction networks use pre-trained models with frozen parameters during training, enhancing the model’s ability to represent complex lesions through feature-level complementarity.

#### Lesion segmentation module

The segmentation module uses EfficientNet-B4 as the feature extraction backbone and constructs a U-shaped encoder-decoder architecture. During the encoder stage, multi-scale feature maps are extracted with dimensions of 128 × 128, 64 × 64, 32 × 32, and 16 × 16. The decoder employs a Feature Pyramid Network (FPN) to perform feature fusion. This is achieved through a series of decoding blocks consisting of multiple convolutional layers, GroupNorm, ReLU, and bilinear upsampling, which progressively decode the fused encoder features. The feature map resolution is unified to 128 × 128, and the decoder features from each level are fused via channel-wise addition. The final output layer applies a 3 × 3 convolution followed by bilinear upsampling to generate a binary segmentation mask of 512 × 512 pixels.

#### Multi-task classification module

The classification module receives the lesion mask output from the segmentation module and performs hierarchical diagnosis through the following steps: Lesion Localization: The binary mask is converted into a minimal bounding box, retaining aspect ratios within the range of [0.5, 2]. The lesion region is then cropped from the original image and resized to 384 × 384 using cubic interpolation. Feature Extraction: The cropped image is input into two networks: Efficient-Net to B4 (with frozen parameters) and MedImageInsight. The Efficient-Net to B4 model, which is the encoder part of the segmentation module, outputs a 1792-dimensional vector after global average pooling from stage 7. The MedImageInsight model, which only uses the image encoder module with frozen parameters, generates a 1024-dimensional medical feature vector. Both feature sets are reduced to 64 dimensions through a fully connected layer. Feature Fusion: The features from the dual pathways are L2-normalized and concatenated into a 128-dimensional joint feature vector. Hierarchical Classification: The concatenated features are passed through a fully connected layer to perform binary classification. Depending on the task, separate models are trained for cancer vs. non-cancer classification and for high-grade vs. low-grade malignant tumor classification.

#### Training strategy

The system adopts a staged training strategy to enhance stability. Initially, the segmentation module is trained. After completion, the parameters of the feature extraction components are frozen. Subsequently, separate models for cancer/non-cancer classification and high-grade/low-grade classification are trained. Each training phase employs a diversified augmentation strategy to improve model robustness, including:(1) Geometric Transformations: Random scaling (ranging from 0.8 to 1.2), rotation (±20°), translation (±10%), and mirror flipping. (2) Photometric Adjustments: Contrast-limited adaptive histogram equalization (CIAHE) and contrast perturbation to enhance fine-grained features. (3) Noise Injection: Simulated endoscopic imaging defects through Gaussian noise (σ ≤ 15) and median blur (kernel size ranging from 3 to 5). (4) Elastic Deformation: Elastic deformations (α = 0 to 5) to introduce realistic tissue deformations. All augmentations are randomly combined (1–4 types) and applied in sequence. The pixel values are preprocessed via *Z* score normalization with μ = [0.485, 0.456, 0.406] and σ = [0.229, 0.224, 0.225].

The segmentation network utilizes a composite loss function and dynamic optimization strategy: the combination of dice loss (weight = 0.8) and class-weighted cross-entropy (weight = 0.2) addresses the data imbalance caused by the small proportion of lesions in cystoscopy images.^24^ The Adam optimizer (β_1_ = 0.9, β_2_ = 0.999) is used with an initial learning rate of 3e-4. The learning rate is gradually decayed to 1e-5 over 50 epochs using cosine annealing. Early stopping is triggered if the Dice coefficient on the validation set does not improve for 10 consecutive epochs, and the best-performing segmentation model is retained.

During the classification phase, the feature extraction parameters of EfficientNet-B4 (from the segmentation module encoder) and MedImageInsight are frozen. Only the non-segmentation encoder portion of EfficientNet-B4, the feature reduction layer, and the classification layer are optimized. The SGD optimizer (momentum = 0.9, weight decay = 5e-4) is used with a fixed batch size of 24, and the training is conducted for 30 epochs with an initial learning rate of 0.1. This learning rate is decayed to 1e-4 over 30 epochs using cosine annealing. The loss function is a weighted combination of focal loss (weight = 0.5) and cross-entropy loss (weight = 0.5).^25^ Weight sampling is applied during training to balance class distribution by increasing the sampling weight for underrepresented categories.

#### The auxiliary effect of AINCDS

We randomly generated 9 test groups, with each group consisting of 50 benign lesions and 50 malignant lesions randomly selected from dataset. These groups were randomly assigned to three groups of urologists, each comprising 3 urologists with 1–3 years of experience, 3 with 4–10 years of experience or 3 with over ten years of experience. Each urologist independently classified the images and recorded any misclassified images. Then, the test groups assigned to each group of urologists were randomly distributed within the group, ensuring that no urologist assessed the same test group. They subsequently diagnosed bladder cancer with the assistance of AINCDS. If the initial diagnosis was incorrect but the second diagnosis was correct, it was considered as “Additionally Diagnosed with assistance of AINCDS”. We then generated another set of 9 test groups, each comprising 25 high-grade tumors and 25 low-grade tumors, and repeated the aforementioned process. None of the 9 urologists had knowledge of the pathological results of these images, nor were they informed of the composition of the test sets, and no one else was involved in other aspects of this study.

### Quantification and statistical analysis

We use accuracy, sensitivity, and specificity as the main metrics to evaluate AINCDS. Additionally, we will assess system performance through DSC, IOU, recall, precision, PPV, NPV, and F-score. The system performance will be visually represented through ROC curves and PR curves. All statistical analyses were conducted using Python 3.8.17. (Zip S2. Date analysis).

## Supplemental information

Document S1. Tables S1–S3

Data S1. Comparison Results of AINCDS and Other Models

Data S2. Data analysis

Data S3. Related code
