# Supplementary material for: Artificial intelligence diagnostics for bladder tumor identification and grade prediction depend on narrow band imaging cystoscopy
Source: iScience. 2025 Dec 3;29(2):114309. doi: 10.1016/j.isci.2025.114309 (PMC12874131; doi:10.1016/j.isci.2025.114309)
Supplement: Document S1. Tables S1–S3 [file mmc1.pdf]

iScience, Volume *29*

Supplemental information

Artificial intelligence diagnostics for bladder tumor identification and grade prediction depend on narrow band imaging cystoscopy

Yinchao Wang, Hao Liang, Yaozhong Zhang, Wenqiang Qi, Guangping Wu, Xiaoyi Zhang, Chuanpeng Li, Shouzhen Chen, Jun Chen, and Benkang Shi

Supplementary table 1. Comparative results of image segmentation

Model	Dataset	Dice	IOU	Recall	Precision
UNet	internal validation set	0.7207	0.6136	0.8089	0.7569
	external validation set	0.7080	0.5887	0.8411	0.6884
DeepLabV3	internal validation set	0.8151	0.7408	0.8155	0.9098
	external validation set	0.7757	0.6782	0.8297	0.8074
AINCDS	internal validation set	0.8832	0.8194	0.8990	0.9039
	external validation set	0.8336	0.7401	0.9017	0.8215

Supplementary table 2. Ablation results of diagnosis of balder cancer

Datasets	Model	Accuracy (95%CI)	Sensitivity (95%CI)	Specificity (95%CI)	PPV (95%CI)	NPV (95%CI)	F1 Score (95%CI)
internal validation set	AINCDS	0.919(0.896 to 0.938)	0.912(0.879 to 0.939)	0.927(0.893 to 0.953)	0.937(0.907 to 0.960)	0.899(0.861 to 0.930)	0.925(0.901 to 0.942)
	MedImageInsight	0.865(0.837 to 0.889)	0.854(0.814 to 0.888)	0.877(0.836 to 0.911)	0.892(0.855 to 0.922)	0.835(0.791 to 0.873)	0.873(0.845 to 0.896)
	efficientnet_b4	0.893(0.868 to 0.915)	0.918(0.885 to 0.943)	0.864(0.822 to 0.900)	0.889(0.854 to 0.919)	0.898(0.859 to 0.930)	0.903(0.877 to 0.923)
external validation set	AINCDS	0.931(0.908 to 0.949)	0.937(0.906 to 0.959)	0.923(0.887 to 0.951)	0.937(0.906 to 0.959)	0.923(0.887 to 0.951)	0.937(0.915 to 0.954)
	MedImageInsight	0.829(0.798 to 0.857)	0.882(0.844 to 0.913)	0.766(0.714 to 0.813)	0.821(0.779 to 0.857)	0.842(0.793 to 0.883)	0.850(0.819 to 0.875)
	efficientnet_b4	0.846(0.816 to 0.873)	0.923(0.890 to 0.948)	0.753(0.700 to 0.800)	0.819(0.778 to 0.855)	0.889(0.844 to 0.925)	0.868(0.839 to 0.892)

Supplementary table 3. Ablation results of prediction of tumor grade

Datasets	Model	Accuracy (95%CI)	Sensitivity (95%CI)	Specificity (95%CI)	PPV (95%CI)	NPV (95%CI)	F1 Score (95%CI)
internal validation set	AINCDS	0.764(0.714 to 0.810)	0.832(0.772 to 0.881)	0.656(0.564 to 0.739)	0.795(0.733 to 0.848)	0.708(0.615 to 0.790)	0.813(0.764 to 0.853)
	MedImageInsight	0.739(0.687 to 0.786)	0.694(0.624 to 0.758)	0.811(0.731 to 0.877)	0.855(0.791 to 0.906)	0.623(0.542 to 0.698)	0.766(0.714 to 0.810)
	efficientnet_b4	0.711(0.657 to 0.760)	0.694(0.624 to 0.758)	0.738(0.650 to 0.813)	0.810(0.742 to 0.866)	0.600(0.517 to 0.679)	0.747(0.694 to 0.792)
external validation set	AINCDS	0.749(0.699 to 0.793)	0.894(0.846 to 0.932)	0.500(0.410 to 0.590)	0.753(0.696 to 0.804)	0.736(0.630 to 0.824)	0.818(0.770 to 0.855)
	MedImageInsight	0.645(0.592 to 0.695)	0.743(0.680 to 0.800)	0.477(0.388 to 0.567)	0.707(0.644 to 0.765)	0.521(0.427 to 0.615)	0.725(0.672 to 0.769)
	efficientnet_b4	0.613(0.559 to 0.664)	0.693(0.627 to 0.753)	0.477(0.388 to 0.567)	0.693(0.627 to 0.753)	0.477(0.388 to 0.567)	0.693(0.639 to 0.739)
